# Elinzanetant (NT-814), a Neurokinin 1,3 Receptor Antagonist, Reduces Estradiol and Progesterone in Healthy Women

**DOI:** 10.1210/clinem/dgab108

**Published:** 2021-02-24

**Authors:** Steve Pawsey, Edouard Gregory Mills, Elizabeth Ballantyne, Kirsteen Donaldson, Mary Kerr, Mike Trower, Waljit Singh Dhillo

**Affiliations:** 1 NeRRe Therapeutics Limited, Stevenage, SG1 2FX, UK; 2 Department of Metabolism, Digestion and Reproduction, Imperial College London, London, W12 ONN, UK; 3 Jade Consultants (Cambridge) Limited, CB24 8RX, UK; 4 Imperial Consultants, Imperial College London, London, SW7 2PG, UK

**Keywords:** neurokinin B, substance P, GnRH, estradiol, uterine fibroids, endometriosis

## Abstract

**Context:**

The ideal therapy for endometriosis (EM) and uterine fibroids (UFs) would suppress estrogenic drive to the endometrium and myometrium, while minimizing vasomotor symptoms and bone loss associated with current treatments. An integrated neurokinin-kisspeptin system involving substance P and neurokinin B acting at the neurokinin (NK) receptors 1 and 3, respectively, modulates reproductive hormone secretion and represents a therapeutic target.

**Objective:**

This work aimed to assess the effects of the novel NK1,3 antagonist elinzanetant on reproductive hormone levels in healthy women.

**Methods:**

A randomized, single-blinded, placebo-controlled study was conducted in 33 women who attended for 2 consecutive menstrual cycles. In each cycle blood samples were taken on days 3 or 4, 9 or 10, 15 or 16, and 21 or 22 to measure serum reproductive hormones. In cycle 2, women were randomly assigned to receive once-daily oral elinzanetant 40, 80, 120 mg, or placebo (N = 8 or 9 per group).

**Results:**

Elinzanetant dose-dependently lowered serum luteinizing hormone, estradiol (120 mg median change across cycle: –141.4 pmol/L, *P* = .038), and luteal-phase progesterone (120 mg change from baseline on day 21 or 22: –19.400 nmol/L, *P* = .046). Elinzanetant 120 mg prolonged the cycle length by median of 7.0 days (*P* = .023). Elinzanetant reduced the proportion of women with a luteal-phase serum progesterone concentration greater than 30 nmol/L (a concentration consistent with ovulation) in a dose-related manner in cycle 2 (*P* = .002). Treatment did not produce vasomotor symptoms.

**Conclusion:**

NK1,3 receptor antagonism with elinzanetant dose-dependently suppressed the reproductive axis in healthy women, with the 120-mg dose lowering estradiol to potentially ideal levels for UFs and EM. As such, elinzanetant may represent a novel therapy to manipulate reproductive hormone levels in women with hormone-driven disorders.

Uterine fibroids (UFs) affect up to 25% of women (1) and endometriosis (EM) 10% of women worldwide (2). Physiological levels of estrogen provide a hormonal drive to the endometrium and myometrium and hence pharmacotherapies to lower estrogen levels are effectively used to treat patients with these disorders (3-6). One such approach that is effective in treating patients with UFs and EM is the use of long-acting gonadotropin-releasing hormone (GnRH) agonists or GnRH antagonists to downregulate the reproductive axis and thereby successfully lower estradiol levels (7-12). However, these drugs cause postmenopausal estradiol levels with resultant hot flashes and bone loss (13-16). An ideal therapy would lower estradiol concentrations to reduce hormonal drive to the endometrium and myometrium, but not to the levels that cause the adverse consequences of current treatments. Such a selective approach is possible because different tissues have different responsiveness to estrogen and a target estradiol serum concentration range of 30 to 50 pg/mL (equivalent to 110-184 pmol/L) has been proposed as a level that will be effective in reducing the symptoms of UFs and EM, but not cause hot flashes and bone loss (17, 18).

Novel approaches are needed that could dose-dependently and reliably reduce estradiol levels to the target estradiol concentrations. It is now well established that GnRH secretion is modulated by hypothalamic neurons expressing kisspeptin, neurokinin B (NKB), and dynorphin (the so-called KNDy neurons) (19-26). NKB, encoded by the *TAC3* gene in humans, has been shown to stimulate GnRH neuronal secretion via action at the neurokinin 3 receptor (NK3R, encoded by *TACR3*) (27-36). In keeping with this, NK3R antagonists have been demonstrated to reduce GnRH pulsatility and reduce gonadotropins and estradiol levels in women (37-42). In addition, substance P (SP; encoded by the *TAC1* gene) acting at the NK1 receptor (NK1R, encoded by *TACR1*) has also been shown to stimulate GnRH neuronal activity and luteinizing hormone (LH) release in humans (43), as well as by selective NK1R agonism in mice (44). Therefore, NK1R antagonism may also lead to lowering of gonadotropins and estradiol levels.

Elinzanetant, previously known as NT-814 (45), is a dual NK1,3R antagonist and therefore has the potential to reduce GnRH pulsatility by blocking the endogenous effects of NKB and SP on the reproductive axis. This would lower LH levels and subsequently estradiol concentrations in women. In this clinical study we evaluated the effect of elinzanetant on gonadotropin and estradiol levels in healthy premenopausal women.

## Materials and Methods

### Ethical Approval

Ethical approval for this study was obtained from the Advarra Institutional Review Board (Columbia, Maryland, USA) and participants provided informed written consent. The study was conducted in accordance with the Declaration of Helsinki and International Council for Harmonization guidelines on Good Clinical Practice.

### Participants

Healthy ovulatory women aged 18 to 45 years, with regular monthly menstrual cycles, body mass index of 18 to 32, not taking any medications or hormonal contraception, were invited to take part in the study. All women underwent detailed medical/medication/menstrual history and clinical examination. The following blood tests were assessed during the screening visit to confirm health status: full blood count, renal function, liver function, bone profile, thyroid hormone profile, LH, follicle-stimulating hormone (FSH), estradiol, progesterone, and hepatitis B and C and HIV serology. All women consented to use 2 methods of contraception, one of which was a barrier method with spermicide, for the duration of the study and 30 days after the last dose of study medication. Acceptable methods of contraception included surgical sterilization of the participant’s male partner, a nonhormonal intrauterine device, and barrier methods (such as the male condom or cap, diaphragm, or sponge with spermicide). No participant received a hormonal contraceptive by any route (systemic, implants, depot or intrauterine).

### Sample Size

**Figure 1.**
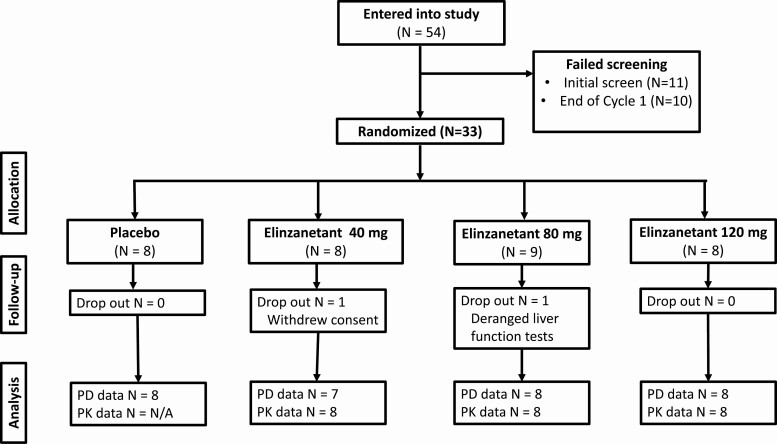
Consolidated Standards of Reporting Trials participant flow depicts the number of participants entered into the study, randomly assigned, and analyzed. Fifty-four women were recruited and screened, of whom 33 were randomly assigned to receive placebo or elinzanetant 40 mg, 80 mg, or 120 mg (N = 8 or 9 per group). The most common reason for screen failure was menstrual period duration or irregularity. One woman in each of the elinzanetant 40-mg and 80-mg groups did not complete the treatment course. A total of 31 participants were included in the pharmacodynamic (PD) per-protocol analyses. Twenty-four participants from the elinzanetant 40-mg, 80-mg, and 120-mg groups were included in the pharmacokinetic (PK) analysis.

Following screening and informed consent, 33 participants took part in the study, resulting in N = 8 or 9 per group (Fig. 1). Two participants did not complete the study (one because of withdrawal of consent and one because of deranged liver enzymes), with one woman in each of the elinzanetant 40-mg and 80-mg groups not completing the treatment course. This sample size is typical of exploratory mechanistic studies and is considered sufficient for the objectives of the study to be achieved.

### Study Protocol

This was a phase 1, randomized, single-blinded, placebo-controlled study designed to determine the effects of elinzanetant on reproductive hormones in healthy women and was performed at a single site (Quotient Sciences, Miami, USA). Thirty-three women participated in the study during 2 consecutive menstrual cycles: cycle 1 (baseline assessment cycle) and cycle 2 (treatment cycle).

**Figure 2.**
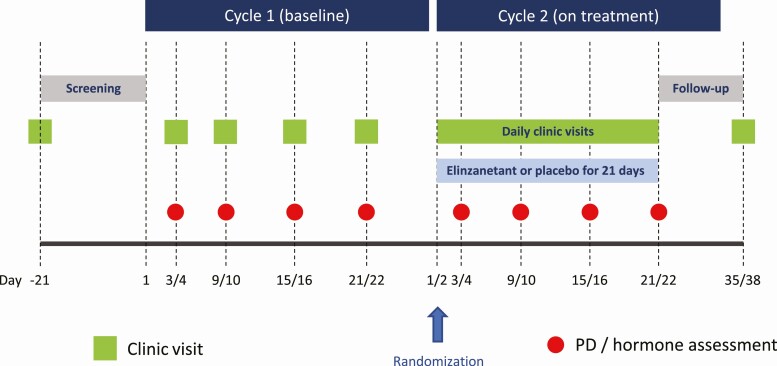
Study protocol diagram. Healthy women attended for 2 consecutive menstrual cycles. In each cycle, blood samples were taken on days 3 or 4, 9 or 10, 15 or 16, and 21 or 22 to measure serum reproductive hormone levels, and plasma elinzanetant levels (cycle 2 only). No treatment was given in cycle 1 (baseline). During cycle 2, participants were randomly assigned to receive placebo or elinzanetant 40 mg, 80 mg, or 120 mg (N = 8 per group [9 for the 80-mg dose]) for up to 21 days.

Women attended the research unit the morning of cycle 1 day 3 or 4 and returned for 3 further outpatient visits the morning of day 9 or 10, day 15 or 16, and day 21 or 22. Serum reproductive hormone levels (LH, FSH, estradiol, and progesterone) were measured at the same time of day at each visit. Women returned the morning of the first day of their next menstrual cycle (cycle 2 day 1) and were randomly assigned to receive elinzanetant 40 mg, 80 mg, 120 mg, or placebo once daily for up to 21 days. Blood samples were collected for serum reproductive hormone levels on cycle 2 day 3 or 4, day 9 or 10, day 15 or 16, and day 21 or 22, with the day and time matching that used during cycle 1. Simultaneous plasma elinzanetant levels were measured in cycle 2 (except for participants in the placebo group). Serum pregnancy tests were conducted at screening and cycle 1 day 21 or 22, and urine pregnancy test on cycle 2 day 1 or 2 (prior to the first dose of study medication). A positive pregnancy test was to result in study withdrawal. Standard safety assessments were conducted throughout, including clinical observations (blood pressure and heart rate), regular 12-lead electrocardiogram evaluation, and clinical laboratory tests. See Fig. 2 for the study protocol.

The study was single-blinded with participants unaware of the treatment group to which they had been allocated. Participants were assigned a subject number in the order they were enrolled and were randomly assigned into 1 of the 4 treatment groups by matching the subject number with the treatment specified by the randomization list generated by Quotient Sciences Biostatistics. Elinzanetant was administered as 40-mg oral soft-gel capsules and was identical in appearance to the placebo capsules. The different doses were provided by giving a mix of placebo and 40-mg capsules to ensure that participants in each of the groups had the same number of capsules to ensure participant blinding. The doses of elinzanetant used were selected based on the known receptor pharmacology and pharmacokinetics of elinzanetant, and those studied in women with menopausal hot flashes (46).

### Hormone Assays

Blood samples were collected at the time points depicted in Fig. 2. Blood samples were analyzed for measurement of serum LH and FSH by immunoassay, and serum estradiol and progesterone using ultrasensitive liquid chromatography with tandem mass spectrometry (Q^2^ Solutions). Reference ranges were as follows: LH in international units per liter (IU/L), 1.9 to 12.5 (follicular), 8.7 to 76.3 (midcycle), 0.5 to 16.9 (luteal); FSH in IU/L, 2.5 to 10.2 (follicular), 3.4 to 33.4 (midcycle), 1.5 to 9.1 (luteal); estradiol in picomoles per liter (pmol/L), 143 to 1377 (follicular), 345 to 2797 (midcycle), 176 to 1615 (luteal), and progesterone in nanomoles per liter (nmol/L) less than 2.2 (early follicular), less than 46.4 (late follicular), less than 51.5 (midcycle), less than 100.2 (luteal). Interassay coefficients of variation were as follows: LH, 7.5%; FSH, 8.2%; estradiol, 2.7%; and progesterone, 7.2%. Limits of detection for each assay were as follows: LH, 0.7 IU/L; FSH, 0.3 IU/L; estradiol, 2 pmol/L; progesterone, 0.1 nmol/L.

Elinzanetant was determined in plasma samples by a validated liquid chromatography with tandem mass spectrometry method (Aptuit). Three analytical batches were run for this study phase. All analytical batches met the acceptance criteria. Average within-run precision for the 3 analytical batches ranged between 4.8% and 6.5%. The limit of quantification was 1.5 ng/mL.

### Statistical Methods

Statistical analyses were performed using the Statistical Analysis System v9.4 (SAS Institute) and GraphPad Prism v7.0e (GraphPad Software). Categorical data were presented using counts and percentages. Parametrically distributed continuous variables were reported as mean ± SD, whereas skewed continuous variables were summarized using median with interquartile range. Parametrically distributed variables were compared using unpaired 2-tailed *t* test (2 groups) or 1-way analysis of variance (multiple groups) with post hoc Tukey. The difference between the change from baseline in hormone levels for each elinzanetant treatment group vs placebo was analyzed using a Hodges-Lehmann estimate of the difference in the medians, and a *P* value calculated using a Wilcoxon rank sum test. A *P* value of less than .05 was considered statistically significant. Both intention-to-treat and per-protocol analyses were undertaken, with the pharmacodynamic data reported using the latter approach. For pharmacokinetic data, all recruited participants who provided at least one quantifiable elinzanetant level were used to calculate the geometric mean. The analyses comparing changes from cycle 1 to cycle 2 in elinzanetant-treated participants with the changes in placebo-treated individuals were prespecified. The within treatment group analyses were undertaken post hoc.

## Results

### Baseline Characteristics

The baseline characteristics are summarized in Table 1. The age range of the women in this study was 19 to 45 years. The individual treatment groups were well balanced for most demographics with no notable differences in age, body weight, and body mass index between the groups.

**Table 1.** Baseline characteristics of participants

Clinical characteristics		Placebo (n = 8)	Elinzanetant 40 mg (n = 8)	Elinzanetant 80 mg (n = 9)	Elinzanetant 120 mg (n = 8)	*P*
Age, y	Median	32.0	35.5	30.0	29.5	.91
	IQR	9.00	4.75	11.00	11.75	
Sex, n (%)	Female	8 (100)	8 (100)	9 (100)	8 (100)	–
Race, n (%)	White	7 (87.5)	7 (87.5)	8 (88.9)	8 (100)	–
	Black or African American	1 (12.5)	1 (12.5)	1 (11.1)	0	–
Weight, kg	Median	69.70	63.65	73.10	59.75	.68
	IQR	12.85	10.45	23.40	17.68	
BMI, kg/m^2^	Median	25.90	24.65	29.20	22.95	.25
	IQR	4.30	2.53	2.60	5.05	
Smoker, n (%)	Nonsmoker	8 (100)	8 (100)	9 (100)	8 (100)	–

Comparison is made using Kruskal-Wallis with post hoc Dunn multiple comparison test.

Abbreviations: BMI, body mass index; IQR, interquartile range.

### Treatment Compliance and Extent of Exposure

Fifty-four women were screened for inclusion in the study, of whom 33 were randomly assigned (see Fig. 1). A total of 31 participants were included in the pharmacodynamic per-protocol analyses, with one woman in each of the elinzanetant 40-mg and 80-mg groups excluded for not completing the treatment course. Twenty-four participants from the elinzanetant 40-mg, 80-mg, and 120-mg groups provided at least one quantifiable plasma elinzanetant level and hence were included in the pharmacokinetic analysis. The median duration of drug exposure in the study was 19.0 days in all 4 treatment groups.

### Effects of Elinzanetant on Serum Luteinizing Hormone Levels

**Figure 3.**
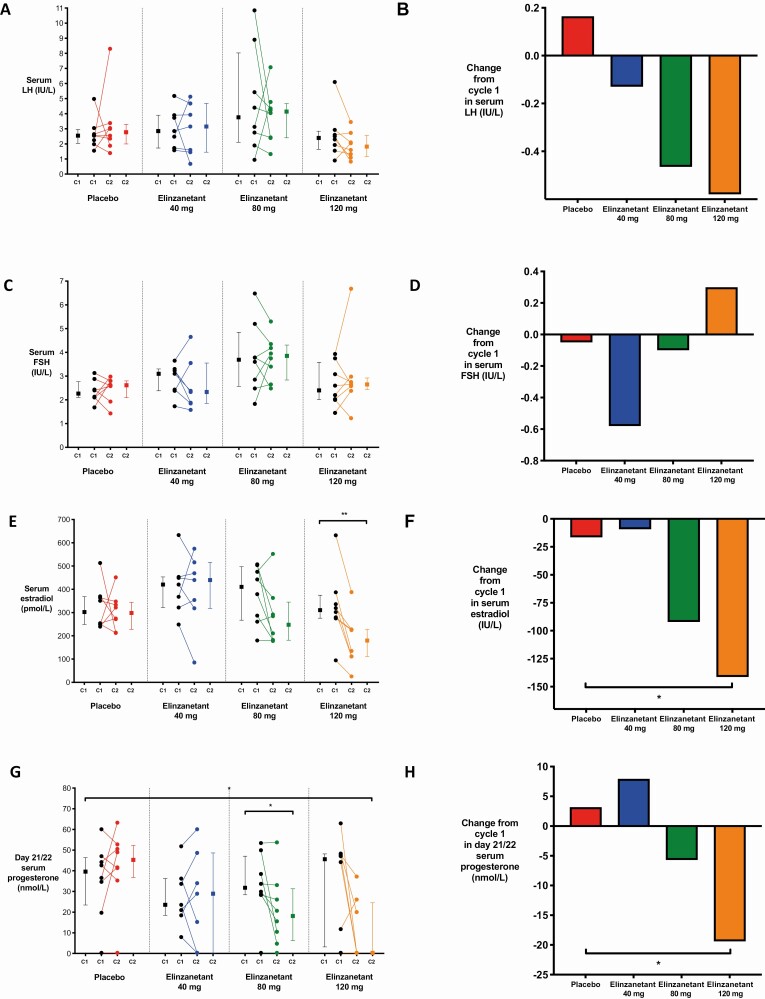
Reproductive hormone levels in women receiving placebo or elinzanetant during the study. A, C, and E, The median averaged across all time points during cycles 1 (C1) and 2 (C2) in A, serum luteinizing hormone (LH; IU/L); C, follicle-stimulating hormone (FSH; IU/L); and E, estradiol (pmol/L) in women after placebo (N = 8, red), elinzanetant 40 mg (N = 7, blue), 80 mg (N = 8, green), and 120 mg (N = 8, orange). Data in graphs depict within-participant paired raw data and the group median and interquartile range. ^**^P less than .01, Wilcoxon matched-pairs signed rank test. B, D, and F, The median change averaged across all time points between cycles 1 and 2 in B, serum LH (IU/L); D, FSH (IU/L); and F, estradiol (pmol/L) in healthy women after placebo (N = 8, red), elinzanetant 40 mg (N = 7, blue), 80 mg (N = 8, green), and 120 mg (N = 8, orange). *P less than .05, Wilcoxon rank sum test. G, The median change from baseline on day 21 or 22 in serum progesterone (nmol/L) in healthy women after placebo (N = 8, red), elinzanetant 40 mg (N = 7, blue), 80 mg (N = 8, green), and 120 mg (N = 8, orange). Data in graphs depict within-participant paired raw data and the group median and interquartile range. *P less than .05, Wilcoxon matched-pairs signed rank test (comparing cycle 1 and cycle 2) and Wilcoxon rank sum test (comparing placebo vs elinzanetant). H, The median change from baseline on day 21 or 22 in serum progesterone (nmol/L) in healthy women after placebo (N = 8, red), elinzanetant 40 mg (N = 7, blue), 80 mg (N = 8, green), and 120 mg (N = 8, orange). *P less than .05, Wilcoxon rank sum test.

Baseline (cycle 1) serum LH levels are summarized in Table 2. As shown in Fig. 3A, a trend toward a dose-dependent reduction in serum LH levels was observed after treatment with elinzanetant. This was reflected in the median of the changes from baseline (average across all time points), which was –0.13, –0.46, and –0.58 IU/L for the elinzanetant 40-mg, 80-mg, and 120 mg groups, respectively, compared with 0.16 IU/L for the placebo group (Fig. 3B), although these differences were not statistically significant (*P* = .69, 0.25, and 0.16, respectively). See Supplementary Fig. 1 for individual participant data for each time point and treatment group (47).

**Table 2.** Median reproductive hormone values (interquartile range) in cycles 1 and 2

Reproductive hormone		Placebo (n = 8)			Elinzanetant 40 mg (n = 7)		
		C1	C2	Change	C1	C2	Change
LH, IU/L	Day 3 or 4	1.90 (2.15)	2.20 (1.63)	0.80	1.80 (1.20)	1.40 (1.00)	0.00
	Day 9 or 10	2.60 (1.38)	2.10 (0.45)	–0.45	2.40 (3.10)	2.90 (8.00)	0.60
	Day 15 or 16	3.40 (2.15)	4.00 (4.45)	0.95	2.50 (4.30)	1.60 (7.60)	–0.80
	Day 21 or 22	1.90 (2.48)	1.35 (1.45)	–0.50	2.30 (2.10)	0.80 (1.10)	–0.50
FSH, IU/L	Day 3 or 4	2.35 (1.50)	2.85 (1.45)	0.75	2.80 (1.00)	2.30 (0.60)	–0.80
	Day 9 or 10	2.15 (1.20)	1.95 (0.75)	0.05	2.90 (1.40)	2.60 (1.30)	0.10
	Day 15 or 16	2.70 (0.68)	2.80 (2.10)	0.30	1.70 (2.00)	1.90 (4.40)	–0.20
	Day 21 or 22	1.70 (0.60)	1.60 (0.88)	–0.10	2.00 (1.50)	1.60 (0.80)	–0.50
Estradiol, pmol/L	Day 3 or 4	101.0 (263.0)	138.0 (95.5)	24.0	136.0 (81.0)	117.0 (139.0)	4.0
	Day 9 or 10	273.5 (186.3)	279.0 (187)	–0.5	334.0 (469)	418.0 (792.0)	–47.0
	Day 15 or 16	271.5 (437.7)	273.5 (437.5)	–18.0	558.0 (401.0)	290.0 (330.0)	–224.0
	Day 21 or 22	284.5 (221.0)	422.5 (116.5)	40.5	444.0 (287.0)	433.0 (404)	–63.0
Progesterone, nmol/L	Day 3 or 4	1.745 (14.710)	1.270 (10.020)	0.000	0.640 (0.320)	0.320 (0.630)	0.000
	Day 9 or 10	0.320 (0.320)	0.320 (0.240)	0.000	0.320 (0.000)	0.320 (0.000)	0.000
	Day 15 or 16	3.815 (8.985)	3.335 (13.280)	–2.065	4.770 (32.430)	17.490 (41.340)	0.000
	Day 21 or 22	39.590 (22.890)	45.315 (15.420)	3.180	23.530 (17.81)	28.940 (48.330)	7.950
Reproductive hormone		Elinzanetant 80 mg (N = 8)			Elinzanetant 120 mg (N = 8)		
		C1	C2	Change	C1	C2	Change
LH, IU/L	Day 3 or 4	1.60 (1.48)	2.35 (2.43)	0.70	1.45 (1.65)	1.20 (1.30)	–0.90
	Day 9 or 10	1.80 (3.25)	2.60 (4.60)	0.15	3.20 (2.48)	1.35 (0.55)	–0.70
	Day 15 or 16	5.60 (22.98)	4.10 (6.33)	–2.40	2.45 (3.28)	2.35 (2.60)	0.60
	Day 21 or 22	1.75 (0.80)	3.20 (3.35)	1.25	1.25 (1.70)	2.10 (1.30)	0.35
FSH, IU/L	Day 3 or 4	2.70 (2.40)	3.35 (1.65)	0.45	3.25 (3.05)	2.25 (0.90)	–1.15
	Day 9 or 10	3.95 (2.65)	3.65 (3.23)	–0.30	2.35 (2.80)	3.40 (1.60)	1.00
	Day 15 or 16	3.75 (6.45)	3.35 (2.33)	–0.55	2.15 (2.00)	2.90 (1.80)	1.05
	Day 21 or 22	2.15 (1.60)	2.50 (2.50)	0.30	1.45 (1.05)	2.25 (1.18)	0.50
Estradiol, pmol/L	Day 3 or 4	147.0 (78.8)	97.5 (69.3)	–20.0	154.5 (131.5)	80.5 (56.5)	–66.0
	Day 9 or 10	215.0 (297.2)	165.0 (136.0)	–38.5	315.5 (214.8)	102.5 (105.8)	–193.0
	Day 15 or 16	562.0 (517.0)	295.5 (349.2)	–187.5	222.0 (147.0)	110.0 (222.3)	–112.0
	Day 21 or 22	406.0 (292.3)	247.5 (276.5)	–112.0	510.0 (414.5)	327.0 (291.7)	–178.0
Progesterone, nmol/L	Day 3 or 4	0.795 (3.653)	0.640 (0.950)	0.000	0.320 (0.240)	0.320 (0.240)	0.000
	Day 9 or 10	0.320 (0.000)	0.320 (0.000)	0.000	0.320 (0.000)	0.320 (0.000)	0.000
	Day 15 or 16	1.430 (13.910)	0.635 (7.075)	0.000	4.390 (12.163)	0.320 (0.713)	–2.860
	Day 21 or 22	31.800 (18.690)	18.125 (25.120)	–5.720	45.630 (44.997)	0.320 (24.25)	–19.400

The median of change from baseline (cycle 1) for each time point was calculated based on the Hodges-Lehmann estimate.

Abbreviations: C1, cycle 1; C2, cycle 2; FSH, follicle-stimulating hormone; IQR, interquartile range; LH, luteinizing hormone.

### Effects of Elinzanetant on Serum Follicle-Stimulating Hormone Levels

Baseline serum FSH levels are summarized in Table 2. As shown in Fig. 3C, a trend toward a dose-related increase in serum FSH levels was observed after treatment with elinzanetant. This was reflected in the median of the changes from baseline (average across all time points), which was –0.58, –0.10, and 0.30 IU/L for the elinzanetant 40-mg, 80-mg, and 120-mg groups, respectively, and –0.05 IU/L for the placebo group (Fig. 3D). However, none of the changes from baseline in FSH in the elinzanetant groups were statistically significantly different to placebo (*P* = .40, .57, and .96 after elinzanetant 40 mg, 80 mg and 120 mg, respectively).

### Effects of Elinzanetant on Serum Estradiol Levels

Baseline serum estradiol levels are summarized in Table 2. There was a marked reduction in serum estradiol levels in the elinzanetant 80-mg and 120-mg groups compared to baseline for all time points, and a reduction in estradiol levels in the elinzanetant 40-mg group compared to baseline on day 15 or 16 (see Table 2). As shown in Fig. 3E, there was a dose-dependent reduction in overall serum estradiol levels (average across the cycle) after treatment with elinzanetant. The median of the changes from baseline was –9.3, –92.1, and –141.4 pmol/L for the elinzanetant 40-mg, 80-mg, and 120-mg groups, respectively, compared with –16.5 pmol/L for the placebo group (Fig. 3F). Following elinzanetant 120 mg, serum estradiol levels were significantly reduced from cycle 1 to cycle 2 (*P* = .008) and the difference compared to placebo was statistically significant (*P* = .038).

### Effects of Elinzanetant on Serum Progesterone Levels

Baseline serum progesterone levels are summarized in Table 2. Following treatment with elinzanetant (cycle 2), there was a dose-dependent reduction in serum progesterone levels during the luteal phase (day 21 or 22) (Fig. 3G). The median of the changes from baseline for day 21/22 was 7.950, –5.720, and –19.400 nmol/L for the elinzanetant 40-mg, 80-mg, and 120-mg groups, respectively, compared with 3.180 nmol/L for the placebo group (Fig. 3H). Following elinzanetant 80 mg, serum progesterone was significantly reduced between cycle 1 and cycle 2 (*P* = .03). Following elinzanetant 120 mg, the reduction compared to placebo was statistically significant (*P* = .046). The overall serum progesterone levels (average across the cycle) and the median changes from baseline after treatment with elinzanetant are summarized in Supplementary Fig. 2A and Supplementary Fig. 2B, respectively (47).

### Effects of Elinzanetant on Menstrual Cycle Length

**Figure 4.**
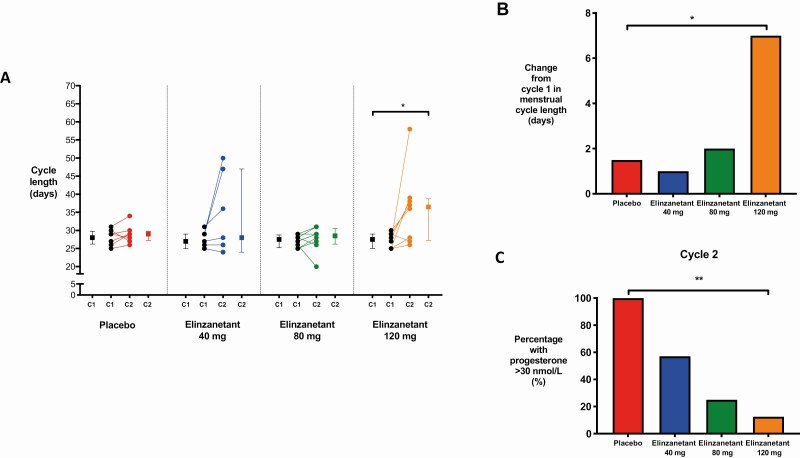
Menstrual cycle length and biochemical parameters of ovulation in women receiving placebo or elinzanetant during the study. A, The change in menstrual cycle length between cycles 1 (C1) and 2 (C2) after treatment with placebo (N = 8, red), elinzanetant 40 mg (N = 7, blue), 80 mg (N = 8, green), and 120 mg (N = 8, orange) in healthy women. Data in graphs depict within-participant paired raw data and the group median and interquartile range. *P less than .05, Wilcoxon rank sum test. B, The median change between cycles 1 and 2 in menstrual cycle length (days) in healthy women after placebo (N = 8, red), elinzanetant 40 mg (N = 7, blue), 80 mg (N = 8, green), and 120 mg (N = 8, orange). *P less than .05, Wilcoxon rank sum test. C, The percentage of healthy women with a serum progesterone greater than 30 nmol/L in cycle 2 after placebo (N = 8, red), elinzanetant 40 mg (N = 7, blue), 80 mg (N = 8, green), and 120 mg (N = 8, orange). ^**^P less than .01, chi-square test.

Median menstrual cycle length in cycle 1 ranged from 27.0 to 28.0 days across the treatment groups (Fig. 4A). Menstrual cycle length increased significantly after treatment with elinzanetant 120 mg by a median of 7.0 days (*P* = .023), with little change observed in the placebo, elinzanetant 40-mg, and 80-mg groups (Fig. 4B).

### Effects of Elinzanetant on Biochemical Parameters of Ovulation

In cycle 1, there was no difference between treatment groups in the proportion of women with a luteal-phase serum progesterone greater than 30 nmol/L (a concentration consistent with ovulation) (48) (*P* = .43). As shown in Fig. 4C, a robust and significant reduction in women with a progesterone greater than 30 nmol/L was observed during treatment with elinzanetant in a dose-dependent manner (*P* = .002).

### Plasma Elinzanetant Levels

Plasma concentrations of elinzanetant, collected at the same time as the hormone samples, showed a dose-ordered increase in exposure, with the 40-mg dose having the lowest concentrations (9.1 ng/mL on day 3 or 4 increasing to 45.3 ng/mL on day 21 or 22) and the 120-mg dose the highest exposure (43.6 ng/mL increasing to 213 ng/mL).

### Safety Assessments

Elinzanetant was well tolerated with no safety concerns identified in this study at doses up to 120 mg once daily for up to 21 days. During the study, there were no clinically relevant changes in symptoms (including an absence of vasomotor symptoms), observations (blood pressure and heart rate), physical examination, or electrocardiogram parameters. There were no notable changes from baseline to follow-up in hematology or clinical chemistry parameters. One adverse event leading to discontinuation of elinzanetant was observed during the study. The participant experienced a mild, nonserious increase in liver enzymes (gamma glutamyl transferase and transaminases) on cycle 2 day 12 (ie, 10 days after the first dose of elinzanetant 40 mg) that resolved following withdrawal of elinzanetant. Detailed examination of the case revealed a past history of elevated liver enzymes and an ultrasound 3 years previously demonstrating fatty liver disease. A repeat liver ultrasound confirmed the presence of fatty liver. Hence, the increased enzymes in this participant were considered by a hepatologist who reviewed the findings to be a coincidental event that was unlikely to be due to elinzanetant. Beyond events expected based on the intended pharmacological effect of elinzanetant and pertaining to delayed menstruation, no significant adverse events were observed.

## Discussion

This is the first report of a dual NK1,3R antagonist (elinzanetant) on reproductive hormones in healthy premenopausal women. Oral administration of elinzanetant (40, 80, and 120 mg once daily) over a full menstrual cycle safely reduced serum LH (albeit statistical significance was not achieved), estradiol, and progesterone levels (particularly during the luteal phase) in a dose-dependent manner, with no plateau of effect observed. Furthermore, a dose-related (but nonsignificant) increase in serum FSH was observed. Moreover, the proportion of women with a serum progesterone level consistent with ovulation was significantly reduced during treatment with elinzanetant in a dose-dependent manner, thus resulting in the observed reductions in median progesterone. Although it is not possible to definitively determine using a single midluteal progesterone value (49-52), this suggests that the proportion of women who became anovulatory in cycle 2 increased in a dose-dependent manner. Cycle length was significantly increased after elinzanetant 120 mg, which likely reflected the changes in LH, estradiol, and progesterone. In this regard, elinzanetant had the anticipated effects of NK1R and NK3R antagonism on reproductive hormone secretion.

Our data are consistent with the proposed mechanism of action of elinzanetant, whereby it acts centrally to modulate pulsatile GnRH secretion by decreasing the GnRH pulse frequency and subsequently reduces downstream reproductive hormones. It is interesting to note that in our study, a minor and dose-dependent increase in serum FSH was also observed. While these changes should be interpreted with caution (statistical significance was not achieved and the frequency of blood sampling may have missed subtle changes in the secretory profile), they are consistent with findings from other clinical studies examining the effects of NK3R antagonism in healthy women (40), although it should also be noted that other groups have identified no effect on FSH secretion from NK3R antagonism (39). Mechanistically, it is well recognized that low GnRH pulse frequency preferentially stimulates FSH secretion, whereas high GnRH pulse frequency favors LH secretion over FSH (53). A reduction in GnRH pulse frequency may explain the minor FSH changes identified in the present study. Moreover, it is plausible that the reduced estradiol resulted in less of an inhibitory effect on FSH secretion (54), resulting in the dose-related increase in FSH. Whether the hormonal effects of short-term elinzanetant administration observed in this study will be maintained with chronic administration or whether compensatory changes will develop over time is currently unknown and will be the focus of future studies exploring chronic elinzanetant administration in this population.

The observed reduction in estradiol levels on all days during cycle 2 with elinzanetant 80 and 120 mg has clear therapeutic implications for hormonally responsive conditions such as EM and UFs, the medical management of which relies on downregulation of the hypothalamic-pituitary-gonadal axis. One approach includes the use of GnRH agonists and antagonists to induce a hypogonadotropic hypogonadal environment (4, 9). However, this is also accompanied by undesirable short-term hypoestrogenic and vasomotor symptoms, as well as long-term effects on bone health and cardiovascular risk, rendering these agents an undesirable long-term strategy. The estrogen threshold hypothesis posits that maintaining estradiol levels within a therapeutic window of 30 to 50 pg/mL (equivalent to 110-184 pmol/L) suppresses estrogenic drive to endometriosis and fibroid cell growth while simultaneously minimizing vasomotor symptoms and bone loss (17, 18). In the present study, this desired range was achieved for part of the cycle with elinzanetant 120 mg, including the cycle average (median 179.8 pmol/L). Therefore, elinzanetant may offer a novel therapy to achieve the desired reduction in hormonal drive to the endometrium or myometrium without increasing bone turnover. Studies in postmenopausal women with hot flashes have demonstrated that elinzanetant 120 mg once daily is a maximally effective dose for this indication (55), but given that no plateau of effect was observed with elinzanetant up to 120 mg once daily in the present study, the effect of higher doses on estrogen levels warrants further evaluation.

The results from the present study are consistent with earlier investigation of NK3R antagonism on reproductive hormone secretion in healthy women. Administration of MLE4901 in the early and mid–follicular phase resulted in reduced LH secretion, prevented follicle growth and rising estradiol secretion, and delayed ovulation by the duration of treatment (38). In addition, MLE4901 delayed the postovulatory progesterone increase without affecting luteal function (38), whereas in the present study elinzanetant dose-dependently lowered progesterone levels. Similarly, fezolinetant (ESN364) taken for 21 days from cycle day 3 dose-dependently decreased basal LH, estradiol, and progesterone levels, resulting in delayed ovulation, decreased endometrial thickening, and prolongation of the menstrual cycle (39). While these studies observed modulation of reproductive hormone secretion, the present study is the first to examine the effect of dual NK1,3R antagonism on reproductive hormones.

Owing to nonequivalence in experimental protocols and the participants examined, caution should be applied when directly comparing the results from the present study with precedent studies of NK3R antagonism. However, in addition to a possible reduction in pain (56, 57), antifibrotic (58), and antiproliferative effects (59), there are theoretical advantages of the dual mechanism of NK1,3R antagonism on reproductive hormone secretion. Indeed, in gonadectomized male and female rats, simultaneous blockade of the 3 NK receptors by administration of CS-003 (a triple NK receptor antagonist) has been observed to suppress LH secretion (60). In the same study, selective antagonists of NK1, NK2, or NK3 receptors were less efficacious when each antagonist was singly administered in gonadectomized females (60). Furthermore, whereas SP preferentially binds to NK1R, NKA to NK2R, and NKB to NK3R, it is now widely accepted that significant cross-reactivity between NKs and their receptors exist, such that each one is capable of eliciting responses from all 3 NK receptors (61-63). Because multiple NK receptors are involved in generating pulsatile GnRH/LH secretion, it is logical to propose that agents targeting multiple NK receptors (such as elinzanetant) may be more potent in modulating reproductive hormone secretion.

The results of the present study combined with a wealth of experimental data provide converging mechanistic and functional evidence for the role of NKs in GnRH release. Whereas NKB undoubtedly plays a key role (64), substantial data are emerging that also implicate SP as an additional regulator of the reproductive axis. In rats, rabbits, and healthy men, administration of SP has been documented to have a robust stimulatory effect on LH release without modifications in FSH secretion (43, 65, 66). By comparison, administration of an NK1R antagonist (RPR 100893) significantly reduced the amplitude and duration of the preovulatory LH surge in cynomolgus monkeys without affecting the FSH surge (67). Furthermore, robust stimulatory actions from SP on arcuate kisspeptin neurons has been observed in electrophysiological studies of mice (68), as well as SP immunoreactivity detected within kisspeptin and NKB neurons in the human infundibular region (69). Finally, a recent study generated complete NK-deficient female mice (*Tac1/Tac2* gene knockout), resulting in a complete absence of the preovulatory LH surge (70). Intriguingly, a similar phenotype was also identified in wild-type females treated with either NKB or SP receptor antagonists (70). Collectively, these findings implicate SP as an important component of an integrated NK-kisspeptin system that controls gonadotropin release. Hence, NK1R antagonism in combination with NK3R antagonism (as offered by elinzanetant) together serve as an ideal target to lower gonadotropins and estradiol levels.

Strengths of this study included its randomized, single-blinded, placebo-controlled design, as well as standardized assessments and procedures. The study also benefited from good compliance to the protocol and an extremely low withdrawal rate. Only 2 participants (1 due to withdrawal of consent and 1 due to deranged liver enzymes) did not complete the treatment course. As such, whereas both a per-protocol and intention-to-treat analyses were conducted, the per-protocol results are presented. This is the norm in exploratory studies that aim to measure the effects of an intervention under experimental conditions (71).

However, the study is not without limitations. The number of participants was small, the frequency of sampling was limited, and the investigatory duration was short. This sample size is considered typical of preliminary, proof-of-concept studies and is considered adequate to enable the study objectives to be achieved. It is also notable that most participants were White, and the selection criteria may have produced a less generalizable study population. In addition, the study was undertaken in a single center, which could represent a potential source of bias. Finally, the hormone data were influenced by the sample frequency, which occurred at 4 time points in the menstrual cycle. In particular, LH has a rapid and transient peak, therefore the granularity of the LH data may not be sufficient to fully demonstrate the effects of elinzanetant on LH because peak levels may not have been observed in individual participants.

Despite these limitations, the overall study design was sufficient to enable an exploratory assessment of the effect of elinzanetant on reproductive hormone secretion and menstrual cycle length in healthy women before progressing to larger and longer studies, including a detailed assessment of hormone pulsatility. Future studies may also include imaging assessments to evaluate, for example, endometrial thickening and follicle growth, to further characterize the effects of elinzanetant on other indicators of ovulatory cyclicity. Furthermore, to accurately determine the proportion of women who become anovulatory after treatment with elinzanetant, consecutive cycles need to be examined (including ultrasound assessment of ovulation), allowing for inferences to be made across multiple menstrual cycles. Moreover, given the potential to cause anovulatory cycles, the possibility of unopposed estrogen exposure as a risk factor for endometrial hyperplasia (72) requires further assessment. However, such risk may be low, given that elinzanetant suppresses both estrogen and progesterone.

In summary, this is the first study to examine the effects of the nonhormonal NK1,3R antagonist elinzanetant on reproductive hormones. In healthy women, once-daily administration reduced serum estradiol and progesterone in a dose-dependent manner without causing vasomotor symptoms. The 120-mg dose of elinzanetant lowered estradiol levels to potentially ideal levels for UF and EM treatment. The findings from this exploratory study supports both targeting NK1 and NK3 receptors as a therapy to modulate reproductive hormone secretion, as well as further studies with elinzanetant to establish its efficacy and safety in patients with hormone-driven disorders.

## Data Availability

Restrictions apply to the availability of some or all data generated or analyzed during this study to preserve patient confidentiality or because they were used under license. The corresponding author will on request detail the restrictions and any conditions under which access to some data may be provided.

## Abbreviations

EM: endometriosis
FSH: follicle-stimulating hormone
GnRH: gonadotropin-releasing hormone
LH: luteinizing hormone
NK1R: neurokinin 1 receptor
NK3R: neurokinin 3 receptor
NKB: neurokinin B
SP: substance P
UF: uterine fibroid

## Additional Information

***Disclosures:*** S.P., M.K., M.T., and E.B. are employees of and own stock in NeRRe Therapeutics Ltd, which funded and sponsored the study. K.D. and Jade Consultants (Cambridge) Ltd undertakes consultancy for KaNDy Therapeutics. W.S.D. has undertaken consultancy work for Myovant Sciences Ltd and KaNDy Therapeutics. E.G.M. has nothing to disclose.
